# Spectral analysis for pulse rate variability assessment from simulated photoplethysmographic signals

**DOI:** 10.3389/fphys.2022.966130

**Published:** 2022-12-09

**Authors:** Elisa Mejía-Mejía, Panicos A. Kyriacou

**Affiliations:** Research Centre for Biomedical Engineering, City, University of London, London, United Kingdom

**Keywords:** photoplethysmography, pulse rate variability, spectral analysis, fast Fourier analysis, simulation

## Abstract

**Introduction:** Pulse rate variability (PRV) refers to the changes in pulse rate through time and is extracted from pulsatile signals such as the photoplethysmogram (PPG). Although PRV has been used as a surrogate of heart rate variability (HRV), which is measured from the electrocardiogram (ECG), these variables have been shown to have differences, and it has been hypothesised that these differences may arise from technical aspects that may affect the reliable extraction of PRV from PPG signals. Moreover, there are no guidelines for the extraction of PRV information from pulsatile signals.

**Aim:** In this study, the extraction of frequency-domain information from PRV was studied, in order to establish the best performing combination of parameters and algorithms to obtain the spectral representation of PRV.

**Methods:** PPG signals with varying and known PRV content were simulated, and PRV information was extracted from these signals. Several spectral analysis techniques with different parameters were applied, and absolute, relative and centroid-related frequency-domain indices extracted from each combination. Indices from extracted and known PRV were compared using factorial analyses and Kruskal-Wallis tests to determine which spectral analysis technique gave the best performing results.

**Results:** It was found that using fast Fourier transform and the multiple signal classification (PMUSIC) algorithms gave the best results, combined with cubic spline interpolation and a frequency resolution of 0.0078 Hz for the former; and a linear interpolation with a frequency resolution as low as 1.22 × 10^−4^, as well as applying a fifth order model, for the latter.

**Discussion:** Considering the lower complexity of FFT over PMUSIC, FFT should be considered as the appropriate technique to extract frequency-domain information from PRV signals.

## Introduction

Pulse rate variability (PRV) describes the changes in pulse rate (PR) through time when it is measured from pulsatile signals such as the photoplethysmogram (PPG) (Mejía-Mejía et al., 2020). PRV has been proposed as an alternative to heart rate variability (HRV), which refers to the changes in heart rate (HR) through time, and is obtained from electrocardiograms (ECG) (Task Force of the European Society of Cardiology and The North American Society of Pacing and Electrophysiology, 1996; Schäfer and Vagedes, 2013; Mejía-Mejía et al., 2020). PRV has become more popular recently, mainly due to the widespread use of PPG sensors in wearable devices, and to the non-invasive, cost-effective and non-intrusive nature of acquiring PPGs (Kyriacou, 2021).

PRV and HRV originate from the same physiological process, i.e., the autonomic regulation performed on the sino-atrial node, which controls the pumping rate of the heart (Rangayyan, 2002; Shaffer and Ginsberg, 2017). In fact, HR and PR have been shown to be good surrogates (Schäfer and Vagedes, 2013). However, the relationship between HRV and PRV is not entirely understood, and although they show similar trends, there is evidence of differences between these two variables, especially when measured from non-healthy, non-resting or elderly subjects (Schäfer and Vagedes, 2013; Mejía-Mejía et al., 2020).

Two hypotheses have been proposed to explain these differences. Some authors argue that the differences between HRV and PRV are mainly explained by physiological aspects. It has been observed that stress and diseases affect PRV in a different way than HRV (Giardino et al., 2002; Charlot et al., 2009; Khandoker et al., 2011; Mejía-Mejía et al., 2021), whereas other aspects such as pulse transit time, external forces on the arteries and the different nature of ECG and PPG have also been proposed as physiological differences that may explain the dissimilarity between HRV and PRV (Gil et al., 2010; Trajkovic et al., 2011; Chen et al., 2015). These and the fact that PRV has been observed in the absence of HRV (Constant et al., 1999; Pellegrino et al., 2014) suggest that there are different processes affecting PRV that are not related to HRV.

An alternative hypothesis is that the agreement between HRV and PRV is affected by technical aspects when PRV is extracted from pulsatile signals (Posada-Quintero et al., 2013; Hemon and Phillips, 2016; Choi and Shin, 2017; Béres et al., 2019; Mejía-Mejía et al., 2022). This is a particularly crucial issue, considering that there are no published guidelines for the extraction of PRV from pulse waves and the standardisation of the related analyses. Therefore, most methodologies for PRV studies are based on the guidelines for HRV assessment from ECG signals (Task Force of the European Society of Cardiology and The North American Society of Pacing and Electrophysiology, 1996). Moreover, most studies performed to understand the effects of technical aspects on PRV are based on the comparison between PRV and HRV, which might introduce further biases since PRV is affected differently to HRV by certain physiological processes.

Most studies related with PRV have been based on the extraction of frequency-domain indices, due to their known relationship with sympathetic and parasympathetic activity (Shaffer and Ginsberg, 2017; Mejía-Mejía et al., 2020). However, there is a lack of understanding of how different spectral analysis techniques can affect the obtained results, although it is known that classical and modern approaches for spectral analysis deliver different results and can be affected by several parameters, such as sampling rate, the number of data points used for computing the spectra and the order of the model (Semmlow and Griffel, 2014). Moreover, interbeat intervals are not evenly sampled, which implies that the trends need to be interpolated in order to have an evenly-sampled time-series to which classical and modern methods can be applied to (Clifford, 2006). However, the effects of this interpolation on the measured spectra and the related indices is not clear, and there is no standard approach to apply this interpolation to the data. The aim of this study was then to determine the best combination of parameters for the extraction of frequency-domain indices from PRV, in a first attempt to establish guidelines for the extraction of frequency-domain information from PRV trends. In this first study, this was done using PRV trends extracted from simulated PPG signals with simulated PRV information, which was considered as gold standard. The main advantages of using simulated signals were 1) the availability of larger amounts and more heterogeneous data, and 2) the comparison of obtained results to a known gold standard rather than HRV, which could introduce additional errors and physiologically-induced differences.

## Materials and methods

The aim of this study was to determine the best combination of parameters for the extraction of frequency-domain indices from PRV, considering PPG signals simulated with a properly selected sampling rate and applying the best performing combination of inter-beat intervals (IBIs) detection algorithm and fiducial points (Mejía-Mejía et al., 2022). The simulation and processing of photoplethysmographic signals was performed in MATLAB (version 2020b), while statistical analyses were done in RStudio (version 1.4.1717).

### Signal simulation

**FIGURE 1:**
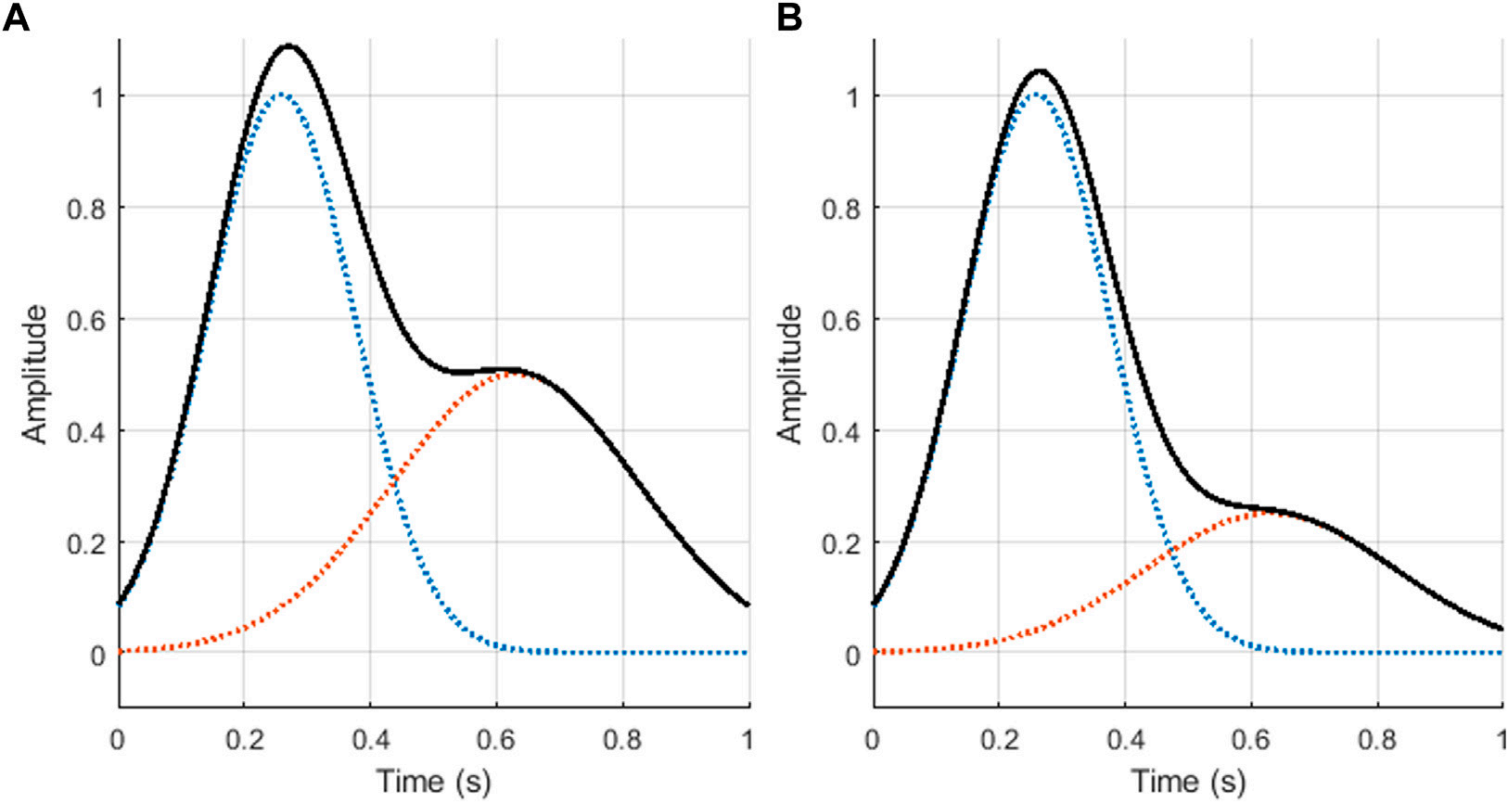
Photoplethysmographic cardiac cycles generated using the proposed mode, using ratios of value **(A)** *r* = 2 (excellent quality), and **(B)** *r* = 4 (acceptable quality). The blue and orange dotted lines illustrate the two Gaussian functions generated, while the black continuous line shows the result of summing these two Gaussian functions, i.e., *z*.

PPG signals were simulated using the model described by Mejía-Mejía et al. (2022). This model is based on the work proposed by Tang et al. (2020b) and Tang et al. (2020a), where each cardiac cycle is simulated using the sum of two Gaussian functions, with parameters set to simulate excellent and acceptable quality PPG signals. The resulting model for a single PPG cycle is shown in Eq. 1, where *θ* corresponds to the four quadrant inverse tangent of the cosine and sine functions of the duration of the cycle; *a*_*i*_, *b*, and *μ*_*i*_ correspond to the height, width and mean values of the Gaussian function; and *r* is a parameter that can be selected to control the relationship of the amplitudes of both Gaussians. This is the main parameter that differentiates between excellent and acceptable quality PPG cycles, and determines the amplitude of the dicrotic notch. In this study, two groups of PPG signals with different values for the *r* parameter were simulated. Excellent quality PPG signals were simulated with ratios of *r* = 2, while acceptable quality PPG signals were considered as those with *r* = 4. Figure 1 shows the base cardiac cycles used for the simulation of excellent and acceptable quality signals.

$$z=a\left(e^{-\frac{{\left(\theta -\mu_{1}\right)}^{2}}{2b_{1}^{2}}}\right)+\frac{1}{r}a\left(e^{-\frac{{\left(\theta -\mu_{2}\right)}^{2}}{2b_{2}^{2}}}\right)$$ (1)

The simulated cardiac cycles were then appended to create a PPG signal with a determined length. The duration of each of the cardiac cycles, i.e., the width of the summation of the Gaussians, was modified in order to include PRV information on the PPG signal. The duration of cardiac cycles was randomly generated by simulating PRV information as a sum of sinusoidal waves with parameters that fall inside plausible physiological values for PRV. The ranges for these parameters are shown in Table 1. It is worth mentioning that this is not the only possible way to generate PRV information, and other models could modify the behaviour of the obtained signal.

**TABLE 1:** Ranges for the Pulse Rate Variability (PRV) parameters and the generation of PRV gold standard values.

Parameter	Range	Units
Low frequency peak location (LF)	0.04–0.15	Hz
High frequency peak location (HF)	0.15–0.40	Hz
Average pulse rate (PR)	40–200	Beats per minute (bpm)
Standard deviation of pulse rate (SD)	0.05–0.08	s

The resulting function for the randomly generated PRV information is shown in Eq. 2. As can be seen, a total of four sinusoidal waves are summed, each of them with different fundamental frequencies, two for each of the main frequency bands in PRV analysis (*LF*(*i*) and *HF*(*i*)). This was done to increase the variability of the frequency spectrum and to alter the area of each of the frequency bands.

$$PRV=PR+\mathrm{SD}\overset{2}{\underset{i=1}{\sum }}\left(\sin\left(2\pi LF\left(i\right)t\right)+\sin\left(2\pi HF\left(i\right)t\right)\right)$$ (2)

**FIGURE 2:**
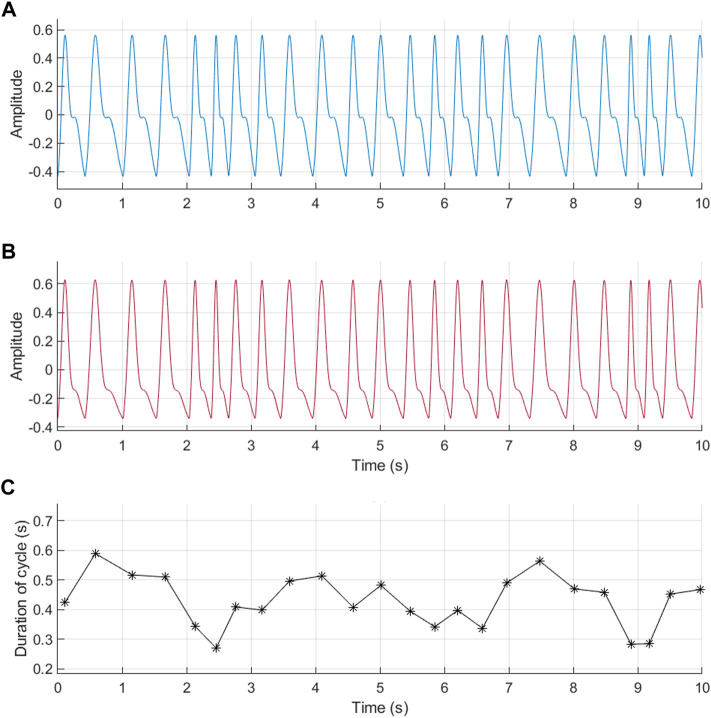
Example of photoplethysmographic (PPG) signals simulated using the proposed model and randomly generated pulse rate variability (PRV) information. **(A)** PPG signal with excellent quality (*r* = 2). **(B)** PPG signal with acceptable quality (*r* = 4). **(C)** PRV information used for the generation of these signals.

For this study, a total of 200 excellent quality and 200 acceptable quality PPG signals were simulated, each with 1,200 cardiac cycles and a sampling rate of 256 Hz. An example of these signals is shown in Figure 2.

### Inter-beat intervals

The cardiac cycles were detected from the simulated signals using the algorithm described by Elgendi et al. ()elg2013, denoted as D2Max, which has been shown to have a good performance for PRV analysis (Mejía-Mejía et al., 2022). This algorithm is based on the generation of blocks of interests based on two moving averages, which are designed based on the expected duration of cardiac cycles and the *a* point in the second derivative of the PPG signal. The location of the systolic peak from the PPG signal is determined as the location of the maximum point in each block of interest.

IBIs were then measured as the time difference between consecutive *a* points detected from each of the identified cardiac cycles. IBIs longer than 1.25 times the median duration of all the IBIs were corrected by looking for additional cardiac cycles in each of these longer windows. IBIs shorter than 0.75 times the median duration of IBIs were also detected and discarded.

### Spectral analysis

Several methodologies for spectral analysis were applied to the extracted IBIs. Fast Fourier transform (FFT) and Welch’s power spectral density (PWELCH) were used as classical methods. For PWELCH, a Hamming window and a 50% overlap between consecutive segments was considered. Yule-Walker’s (PYULEAR), Burg’s (PBURG), covariance (PCOV), and modified covariance (PMCOV) autoregressive models were used to obtain model based methods, as well as the multiple signal classification (PMUSIC) algorithm was used to obtain a pseudo-spectrum. Finally, the Lomb-Scargle algorithm (PLOMB) was also applied. In the case of classical and model-based algorithms, the parameters presented in Table 2 were optimized.

**TABLE 2:** Combinations of parameters used for the extraction of frequency spectra from pulse rate variability trends. Frequency resolution: number of samples used to calculate spectrum (nFFT) divided by the sampling rate of the signal.

Methods	Interpolation		Frequency Resolution (Hz)	Order
	Technique	Sampling rate (Hz)		
Classical	Linear or cubic spline	4, 8, 16, 32, 64, 128, 256	0.01, 0.001, 0.0001	—
Model-based	Linear or cubic spline	4, 8, 16, 32, 64, 128, 256 Hz	0.01, 0.001, 0.0001	5, 10, 15, 20, 25, 30, 35, 40, 45, 50

From the different combinations of parameters and the different methods for spectral analysis, PRV frequency domain indices were extracted. The indices considered in this study were: The power of the very low frequency band (VLF); the absolute and relative power of the low frequency band (LF and nLF); the absolute and relative power of the high frequency band (HF and nHF); the total power of the spectrum between 0.0033 and 0.4 Hz (TP); the ratio between LF and HF (LF/HF); and the coordinates of the centroid of LF, HF and TP (cLF_x_, cLF_y_, cHF_x_, cHF_y_, cTP_x_ and cTP_y_).

These indices were also extracted from the power spectra obtained from the simulated gold standard PRV signals. These were calculated using FFT with 2^16^ number of points (nFFT). Table 3 summarises the PRV indices extracted from gold standard PRV.

**TABLE 3:** Mean and standard deviation (SD) values for PRV indices extracted from gold standard PRV.

Index	Mean ± SD
VLF (ms^2^)	0.0555 ± 0.1430
LF (ms^2^)	1.9781 ± 0.3495
HF (ms^2^)	2.0024 ± 0.3639
TP (ms^2^)	4.0360 ± 0.6787
nLF	0.4925 ± 0.0489
nHF	0.4941 ± 0.0333
LF/HF	1.0078 ± 0.1833
cLF_x_ (Hz)	0.0948 ± 0.0235
cLF_y_(ms^2^)	0.3501 ± 0.0803
cHF_x_ (Hz)	0.2686 ± 0.0463
cHF_y_(ms^2^)	0.3313 ± 0.0864
cTP_x_ (Hz)	0.1799 ± 0.0273
cTP_y_(ms^2^)	0.3410 ± 0.0445

### Statistical analysis

Factorial analyses were performed for each independent spectral analysis method. This was done in order to evaluate the effects of interaction among the studied factors, i.e., type of interpolation used (A), the number of data points used for obtaining the spectrum (B), the sampling rate used for interpolation (C), and the order of the model (D). The difference between the indices extracted from measured and gold standard PRV trends were obtained. These differences were then used for the statistical analysis, in which independent factorial analysis were first performed in order to obtain the combination of factors that gave the lowest differences when spectra was obtained using each of the different methods, except for the Lomb-Scargle periodogram, in which no parameters needed to be modified. Then, the best combination of factors was identified for each of the methods and these were compared using a Kruskal-Wallis test, since data did not follow a normal distribution according to the Lilliefors test of normality of data. Using Wilcoxon rank sum tests with Bonferroni correction, *post hoc* analyses were performed for the indices in which the Kruskal-Wallis analysis showed statistically significant differences among methods. The best combination of method, interpolation technique, frequency resolution and model order was then identified. Since data did not comply with ANOVA assumptions for factorial analyses, Box-Cox transformations were applied for the statistical analyses.

Cross-correlation and Pearson (XC Pearson) and Spearman (XC Spearman) correlation analyses were used to compare the frequency spectra obtained from measured and gold-standard PRV. This was done to assess the similarity among spectra extracted with the different combinations of parameters and with the different methods. The cross-correlation was characterized using the maximum value of cross-correlation found (XC max), and the lag at which this maximum occurred (XC lags). A similar process with factorial analyses was performed with these indices, considering that a maximal cross-correlation was desired.

## Results

As explained, a factorial analysis was performed for each independent spectral analysis method. This was done in order to evaluate the effects of interaction among the studied factors, i.e., type of interpolation used (A), the number of data points used for obtaining the spectrum, which relates to the frequency resolution (B), the sampling rate used for interpolation (C), and the order of the model (D, for model-based approaches). The behaviour of indices extracted from excellent and acceptable PPG signals was generally very similar.

In the case of FFT, the interaction between the three factors (A × B × C) was significant for TP and XC lags, whereas it was significant for HF, XC lags and XC Spearman. For both methods, the interaction between the number of data points and sampling rate used for interpolation (B × C) was the most significant, whereas the interactions between the type of interpolation and the other two factors were non-significant in most cases. Centroid related indices were the less affected by the different factors, showing significance on factor A only for cHF_y_ when measured using FFT and PWELCH, and on factor B for cLF_y_ when measured using PWELCH.

In the case of modern methods the behaviour was not as clear, since each method showed different significant interactions. In the case of PYULEAR and PMUSIC, the interactions between the type of interpolation used, the number of data points and the order of the model (A × B × D), as well as the interactions between the type of interpolation, the sampling rate used and the order of the model (A × C × D) were significant in the majority of the indices, while for PBURG, PCOV, and PMCOV the maximum level of significance for most of the indices was with two-factor interactions.

The best combination of factors that gave the lowest difference for the measurement of each of the PRV indices, as well as those that delivered maximal cross-correlation to gold-standard spectra were determined for each of the methods that allowed the selection of parameters, both for excellent and acceptable quality PPG signals. Once the best combinations were identified for each of the methods, these and the results obtained using the Lomb-Scargle periodogram were compared using a Kruskal-Wallis one-way analysis of variance for each index. Tables 4, 5 summarize these results for PRV obtained from excellent and acceptable quality PPG signals, respectively.

**TABLE 4:** Summary of results obtained from the Kruskal-Wallis one-way analysis of variance and *post hoc* comparisons for pulse rate variability obtained from excellent quality PPG signals. ×: Significant differences. —: Non-significant differences.

Index	Best results	Significant differences							
		FFT	PWELCH	PYULEAR	PBURG	PCOV	PMCOV	PMUSIC	PLOMB
VLF	PMUSIC	—	—	—	—	—	—	—	×
LF	PWELCH	—	—	×	×	×	×	×	×
HF	FFT	—	×	×	×	×	×	×	×
TP	FFT	—	×	×	×	×	×	×	×
nLF	PMUSIC	×	×	—	×	×	×	—	×
nHF	PMUSIC	×	×	×	×	×	×	—	×
LF/HF	PMUSIC	×	×	×	×	×	×	—	×
cLF_x_	PMUSIC	—	—	—	—	—	—	—	—
cLF_y_	PCOV	×	×	—	—	—	—	—	×
cHF_x_	PMUSIC	—	—	—	—	—	—	—	-—
cHF_y_	PYULEAR	×	×	—	×	—	—	—	×
cTP_x_	PMUSIC	×	×	—	—	—	—	—	×
cTP_y_	PMUSIC	×	×	—	—	—	—	—	×
XC lags	PMUSIC	×	×	×	×	×	×	—	×
XC max	PLOMB	×	×	×	×	×	×	×	—
Spearman	PCOV	×	×	—	—	—	—	—	—
Pearson	PWELCH	×	—	—	—	—	—	×	—

**TABLE 5:** Summary of results obtained from the Kruskal-Wallis one-way analysis of variance and *post hoc* comparisons for pulse rate variability obtained from acceptable quality PPG signals. ×: Significant differences. —: Non-significant differences.

Index	Best results	Significant differences							
		FFT	PWELCH	PYULEAR	PBURG	PCOV	PMCOV	PMUSIC	PLOMB
VLF	PMUSIC	—	—	—	—	—	—	—	×
LF	PWELCH	—	—	×	×	×	×	×	×
HF	FFT	—	×	×	×	×	×	×	×
TP	FFT	—	×	×	×	×	×	×	×
nLF	PMUSIC	×	×	—	×	×	×	—	×
nHF	PMUSIC	×	×	×	×	×	×	—	×
LF/HF	PMUSIC	×	×	×	×	×	×	—	×
cLF_x_	PMUSIC	—	—	—	—	—	—	—	—
cLF_y_	PBURG	×	×	—	—	—	—	—	×
cHF_x_	PBURG	—	—	—	—	—	—	—	—
cHF_y_	PYULEAR	×	—	—	—	—	—	—	×
cTP_x_	PMUSIC	×	×	—	—	—	—	—	×
cTP_y_	PMUSIC	×	×	—	×	×	×	—	×
XC lags	PMUSIC	×	×	×	×	×	×	—	×
XC max	PLOMB	×	×	×	×	×	×	×	—
Spearman	PCOV	×	×	—	—	—	—	—	—
Pearson	PWELCH	×	—	—	—	—	—	×	—

**FIGURE 3:**
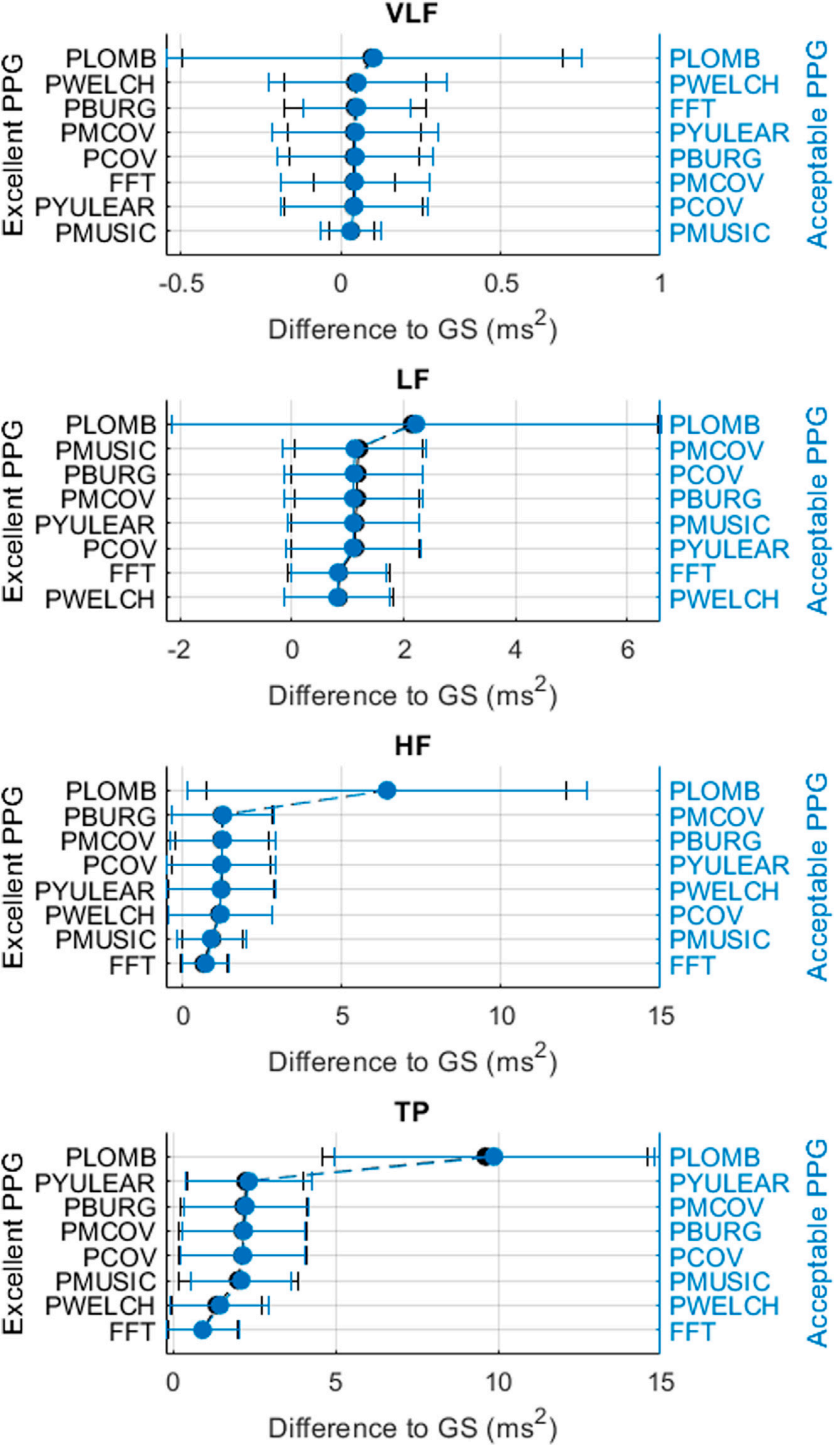
Mean and standard deviations of the differences obtained by comparing pulse rate variability absolute power frequency-domain indices obtained from extracted and gold-standard trends.

**FIGURE 4:**
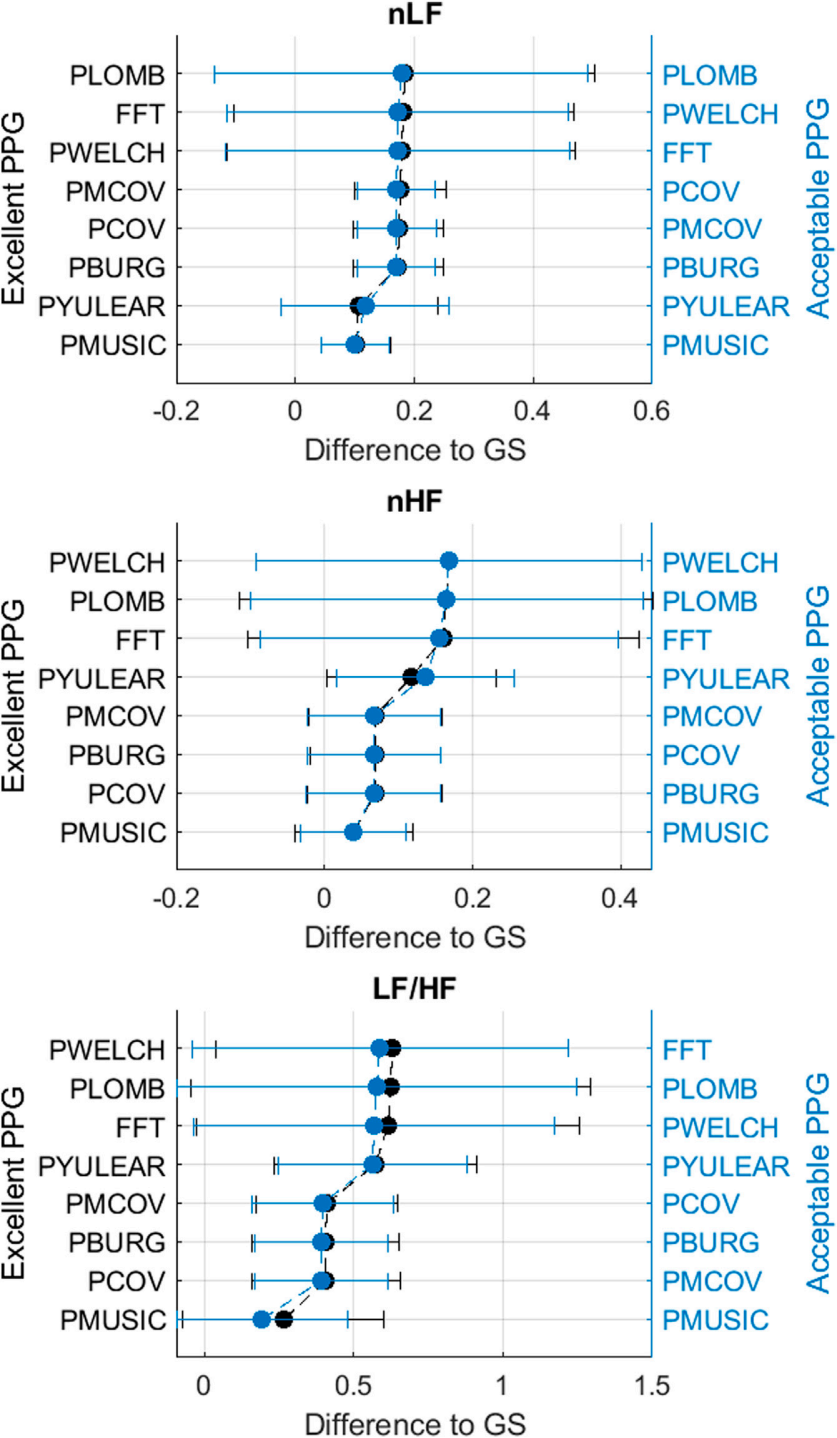
Mean and standard deviations of the differences obtained by comparing pulse rate variability relative power frequency-domain indices obtained from extracted and gold-standard trends.

**FIGURE 5:**
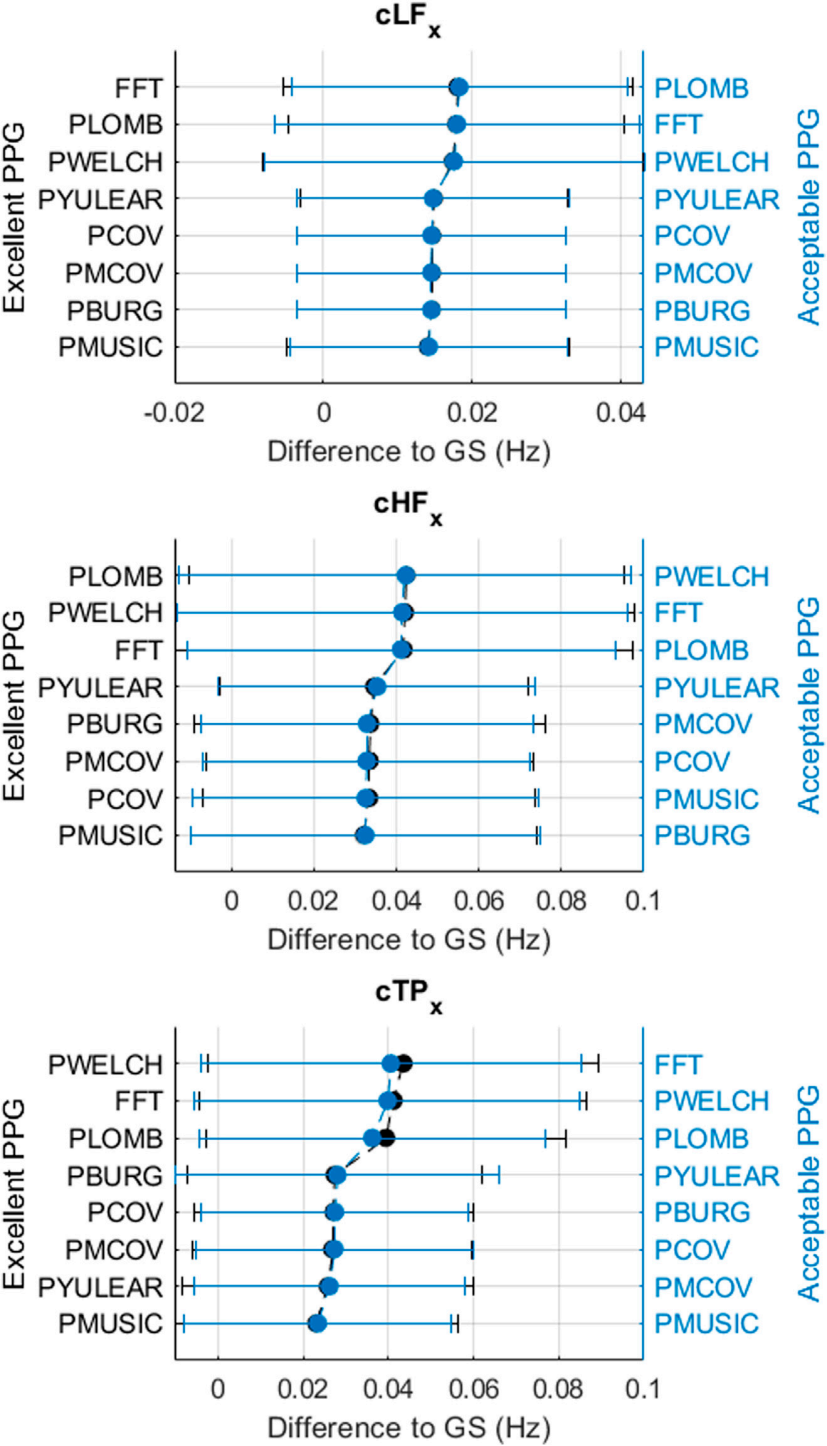
Mean and standard deviations of the differences obtained by comparing pulse rate variability *x*-coordinates of centroid-related frequency-domain indices obtained from extracted and gold-standard trends.

**FIGURE 6:**
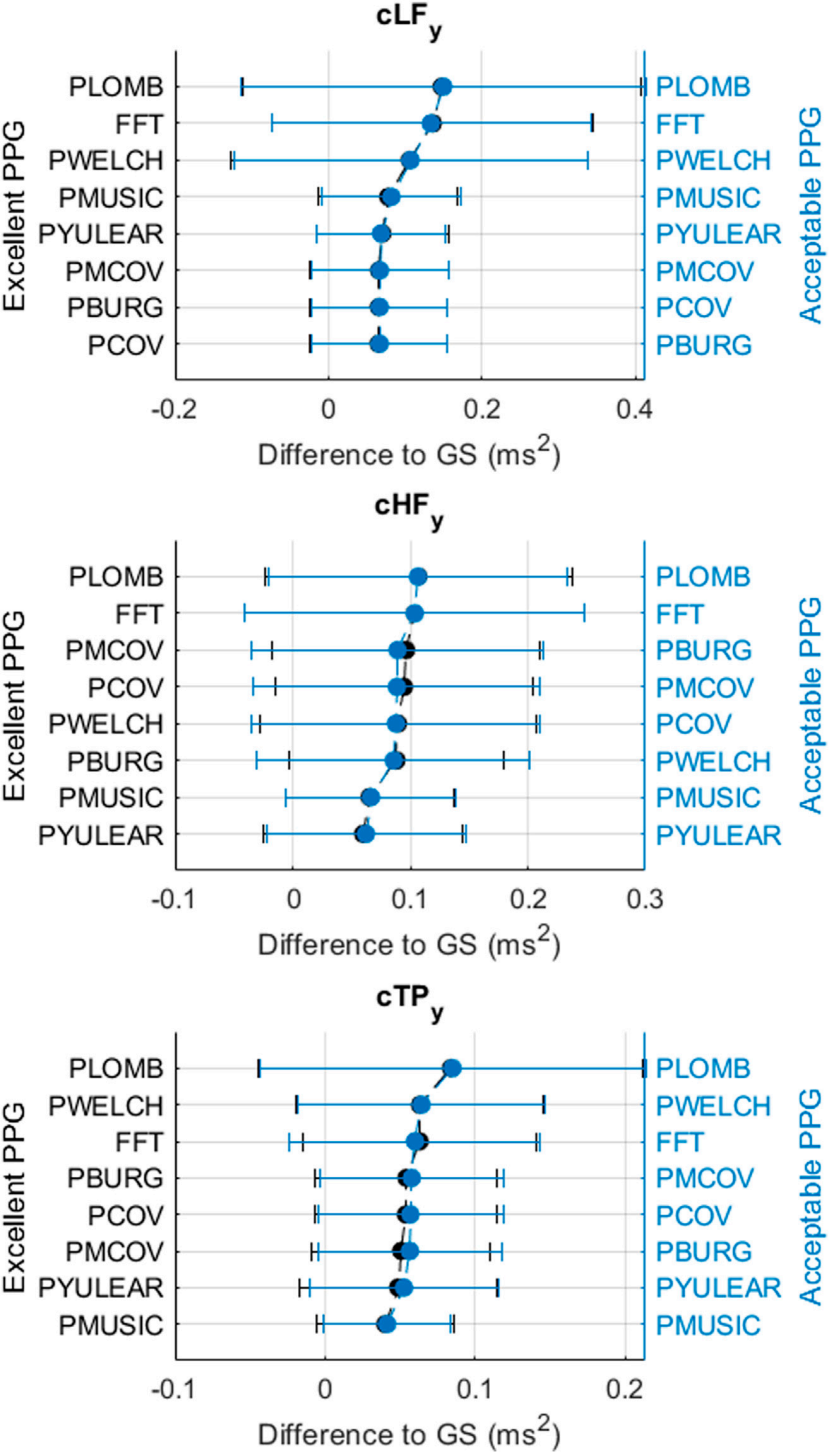
Mean and standard deviations of the differences obtained by comparing pulse rate variability *y*-coordinates of centroid-related frequency-domain indices obtained from extracted and gold-standard trends.

**FIGURE 7:**
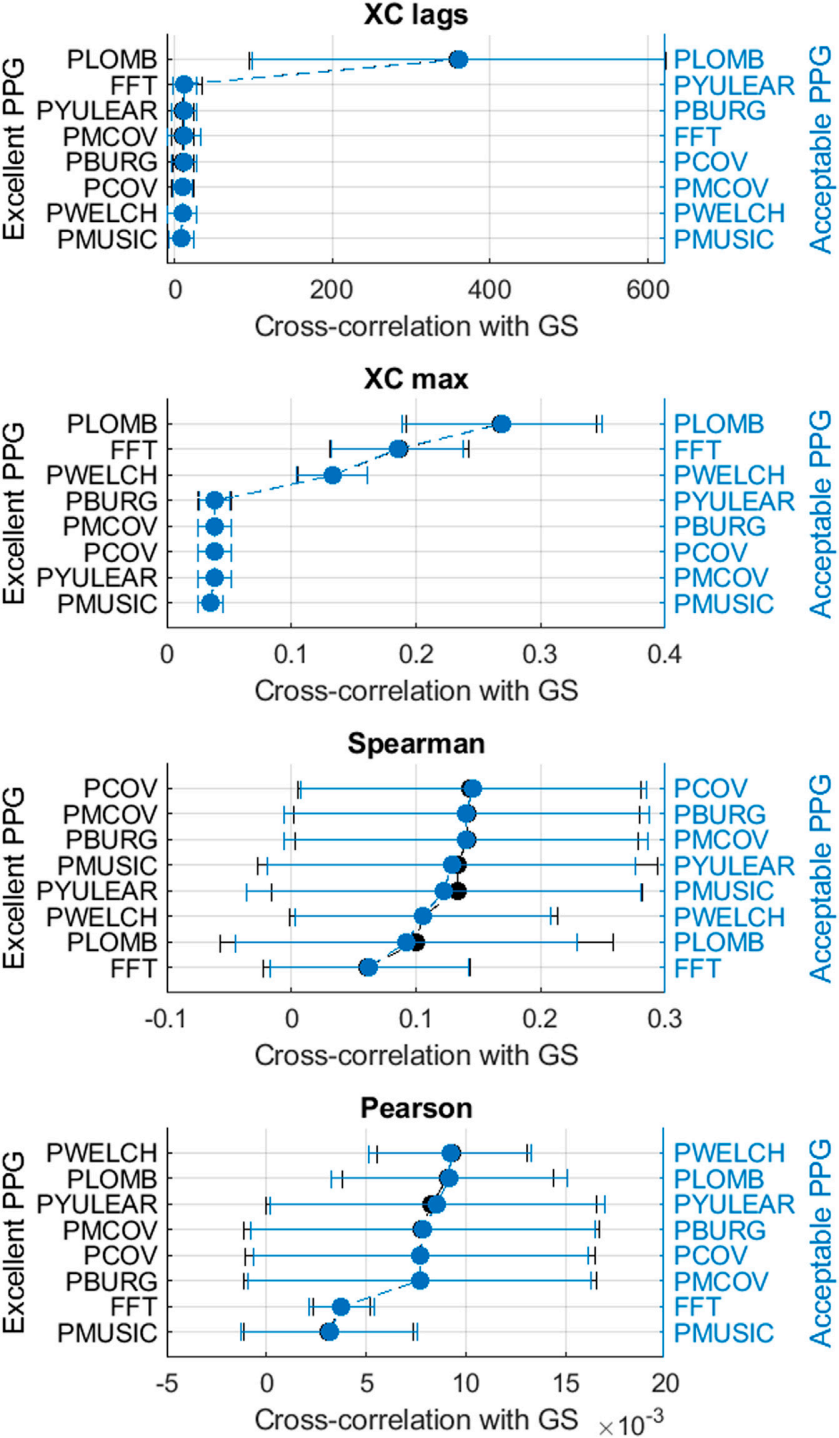
Mean and standard deviations of the correlation results obtained by comparing pulse rate variability spectra obtained from extracted and gold-standard trends.

Figures 3–6 show the mean and standard deviation of the differences of frequency-domain indices obtained between gold-standard and measured PRV trends, considering the best combinations of factors for each spectral analysis method, and Figure 7 summarizes the correlation results after comparing gold-standard and measured PRV spectra. The best spectral analysis should have minimal differences to gold-standard results, while achieving maximal correlation results.

**FIGURE 8:**
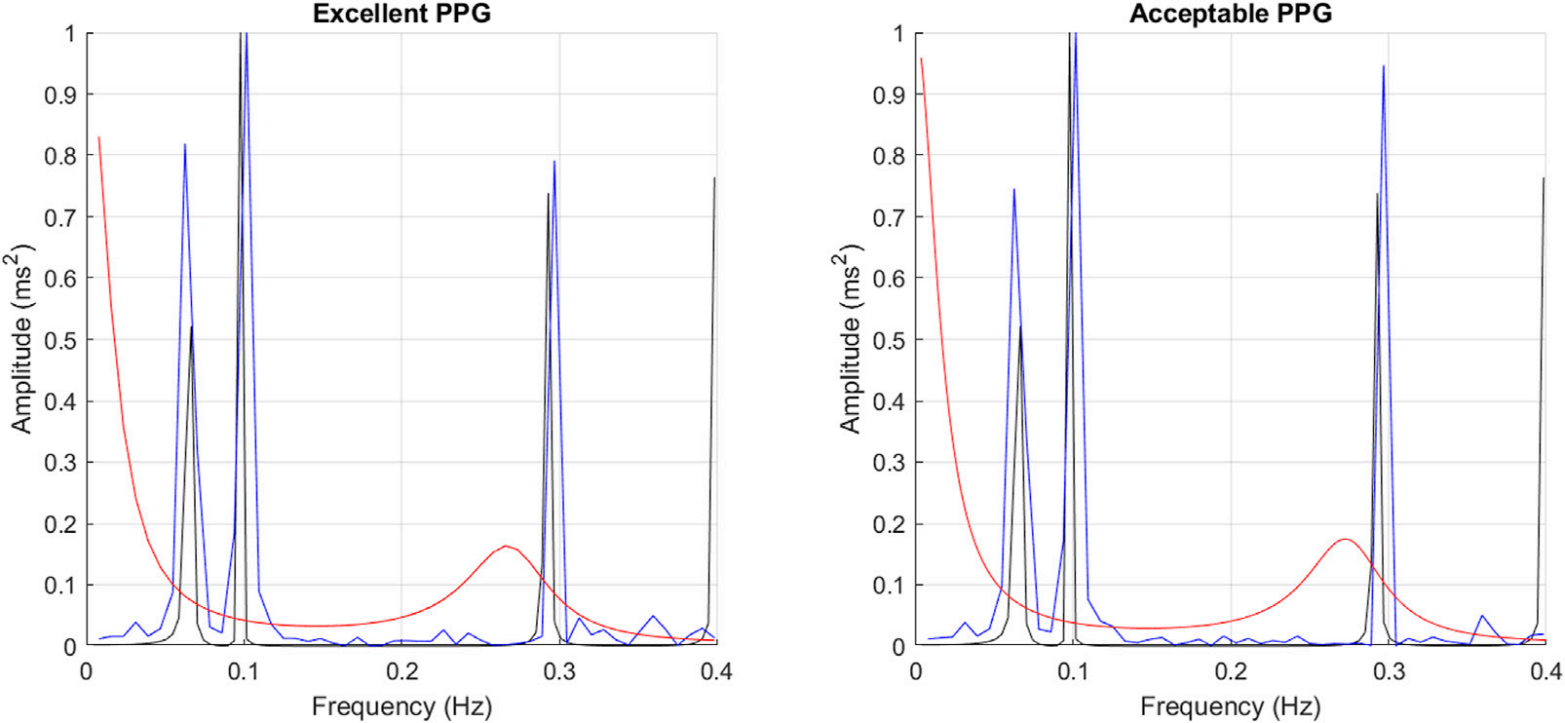
Example of spectra obtained from gold standard PRV (black), FFT (blue) and PMUSIC (red), both from excellent and acceptable quality PPG signals.

It can be observed that the classical method with better performance was FFT, while MUSIC showed the best performance among modern methods. Both for excellent and acceptable quality PPG signals, PMUSIC was the best performing method for 9 and 8 of 17 indices, respectively. In terms of classical methods, FFT showed better behaviour than PWELCH. Also, it was found that the Lomb-Scargle periodogram did not show good reliability for the extraction of frequency-domain indices. Both for excellent and acceptable quality PPG signals, the FFT showed better performances when obtained after applying a cubic spline interpolation and resampling PRV trends to 4 Hz, while an optimal number of samples for measuring the spectrum was 512, which gave a frequency resolution of 0.0078 Hz. In the case of the MUSIC method, resampling PRV trends to 4 Hz using linear interpolation and using a fifth order model gave the best results both for excellent and acceptable quality signals. For excellent quality PPG signals, a resolution frequency of 0.0078 Hz was also found to perform the best, although for acceptable quality PPG signals a number of samples that gave best results increased to 32,768, for a resolution frequency of 1.2207 × 10^–4^ Hz. Figure 8 exemplifies the behaviour of spectra obtained using these spectral analysis techniques and the corresponding parameters. Since FFT algorithm and application is less complex than PMUSIC, and there were not many significant differences between the best combinations of these two methods, applying FFT with the recommended parameters was found to be the best option for PRV spectral analysis.

## Discussion

Frequency-domain indices are probably the most used HRV and PRV features since their relationship with specific processes related to autonomic regulation have been shown in the literature (Task Force of the European Society of Cardiology and The North American Society of Pacing and Electrophysiology, 1996; Billman, 2013; Shaffer and Ginsberg, 2017). However, at least for PRV analysis, there is no consensus regarding how frequency spectra should be derived from PRV time-domain trends, and very little research has been done concerning this issue.

As is mentioned in the guidelines for HRV analysis, the power spectral density (PSD) from HRV can be obtained using non-parametric (classical, such as FFT) and parametric (modern) methods (Task Force of the European Society of Cardiology and The North American Society of Pacing and Electrophysiology, 1996). However, there are multiple algorithms and parameters that can be modified in order to calculate this PSD both from HRV and PRV trends. Also, research related to the optimisation of these parameters and their suitability to obtain frequency domain indices from PRV is scarce, hence reinforcing the need for such research (Li et al., 2019). provided a useful summary of the different methodologies used for spectral analysis from HRV trends. In the case of PRV (Akar et al., 2013), applied several pre-processing techniques for the extraction of PRV indices from PPG signals, and compared the spectra obtained using the periodogram, Welch’s and Burg’s algorithms. Although qualitative, their results showed differences in the extracted spectra due to the methods used for its extraction (Chen et al., 2018). evaluated the differences between frequency-domain indices extracted from PRV trends re-sampled using different sampling rates, concluding that, from data obtained from wearable devices, better results were obtained using a 1 Hz re-sampling rate for interpolating pulse rate information and extracting frequency-related information. Other studies have also suggested the extraction of frequency-related indices using novel time-frequency techniques, such as empirical mode decomposition (Abeysekera and Jaisankar, 2015; Chuang et al., 2015). In this study, the aim was to determine the best parameters for the extraction of spectral information from PRV trends. It was found that the morphology of the spectra, assessed by measuring cross-correlation indices between spectra obtained from gold-standard and measured PRV trends, is affected, in most cases, by all the factors considered for obtaining the PSD and their interaction. However, PRV indices did not show this behaviour. In the case of classical spectral analysis, indices were mostly affected by the number of data points and the sampling rate used for interpolation before extracting PSD. Both these factors are related to the frequency resolution of the obtained spectra, which was shown to be a critical factor for the assessment of frequency-related information regardless of the algorithm used for obtaining the spectra. The comparison of the behaviour of indices extracted using different modern methods is less straightforward, indicating the variability among the mathematical foundations for each of these algorithms. In the case of Yule-Walker and MUSIC algorithms, three-way interactions including the type of interpolation used and the order of the model showed significant behaviour, while for the remaining methods two-way interactions showed the most significant results.

It is noticeable that, in the case of centroid-related indices, there were more significant interactions for indices related to the *y*-coordinate, particularly for the centroid of the high-frequency band. This could be indicating that the different methods for assessing PRV frequency-content tend to be relatively stable for the distribution of the frequency content, but there are differences in terms of the amplitude of the spectra. Hence, additional care should be taken when amplitude-related indices are of interest. Also of interest is the fact that the Lomb-Scargle algorithm did not show a better performance than the other methods studied. This algorithm is based on probability distributions and does not require a periodically-sampled signal (Clifford, 2006). However, its lower performance might be related precisely to the unpredictability of PRV trends, and the largely variable parameters used for the simulation of PRV information.

In general, it was found that MUSIC and FFT had the best behaviour both for excellent and acceptable quality PPG signals. In the case of MUSIC, the best behaviour was found when PRV trends were resampled to 4 Hz using linear interpolation and when a fifth order model was used, both for excellent and acceptable quality PPG signals, with frequency resolution of 0.0078 and 0.000122 Hz respectively. In the case of FFT, the best results regardless of quality of the signal were obtained after applying a cubic spline interpolation to obtain a 4-Hz PRV trend, and calculating the spectrum with 512 data points, for a frequency resolution of 0.0078 Hz. Given the simplicity of FFT, the computational load it has, and the easiness to perform it in any platform, including embedded systems, it is recommended to obtain spectral information from PRV trends using this algorithm and these combination of parameters.

It is important to remark that the gold standard measurements were extracted using FFT, hence a bias could be present due to this. Non-etheless, the fact that the MUSIC algorithm also showed a good performance, and that Welch’s periodogram showed comparable results to FFT, indicate that the results obtained are reliable. Moreover, the improved results obtained using these algorithms can be explained from their theoretical principles. The MUSIC algorithm, which is based on the identification of eigenvalues and eigenvectors from a signal, has been shown to be a high-resolution method particularly suitable for analysing time series that are a sum of sinusoidal waves, such as PRV, contaminated with Gaussian noise (Fernando et al., 2003; Castanié, 2011). In the case of FFT, this is the most direct and efficient digital implementation of the Fourier transform, and hence is a suitable tool for the spectral analysis of sine wave signals (Castanié, 2011; Semmlow and Griffel, 2014). Also, when FFT is compared to other classical methods, it has a higher resolution than other alternatives such as Welch’s method (Semmlow and Griffel, 2014). In general, both MUSIC and FFT could be expected to perform well when the input signal exhibits a sine-like behaviour and when higher resolution is required to observe the behaviour of the signal at lower frequencies, as is the case of PRV.

This study has some limitations. Firstly, simulated PPG signals with simulated PRV information were used in this study. This was done with two main purposes. It is simpler to obtain larger number of samples using simulated data, which gives statistical validity to the experiment. The sample size for this study was estimated to observe differences of 2% in the measurement of the indices, compared to the gold standard. Also, by simulating PRV information it was possible to obtain a gold standard that was not HRV information obtained from the ECG. As mentioned, physiological aspects may explain part of the differences between HRV and PRV, hence comparing them in order to establish methodologies and strategies for obtaining PRV information is not ideal. Regardless of the benefits of using simulated signals, these may not represent the entire variation of the PPG morphology, and the results from these experiments need to be validated using real PPG data. The simulation of PRV information may also affect the results obtained. However, PRV was simulated using physiologically feasible values, which may introduce larger variability of the PRV but also simulate PRV information that could be obtained from most of the healthy population. Future studies should optimise the PRV model to have a better reflection of real PRV information, applying alternative models such as the integral pulse frequency modulation model (Candia-Rivera et al., 2021) or dynamical models such as the one proposed by McSharry et al. (2003). Secondly, the signals simulated were noiseless. This was done to have a controlled way to modify the parameters, but the effect of noise in these results need to be considered in future studies. Also, the agreement between indices was not assessed. Future studies should investigate not only the significance of the difference but also determine how the indices agree using techniques such as Bland-Altman analysis. Finally, the gold standard indices used in this study were extracted from PRV trends using FFT, which could have had a bias on the results. However, this was considered the optimal solution given the response of FFT compared to the rest of the algorithms.

## Conclusion

The relationship between PRV and HRV is not straightforward, both due to physiological differences and to effects of technical aspects on the extraction of PRV information from pulsatile signals such as the PPG. The latter has not been thoroughly studied and there is no consensus regarding the methodologies for the extraction of PRV. In this study, a first approach for determining the best combination of factors for the extraction of frequency-domain indices from PRV information from simulated PPG signals was presented. It was found that spectral analysis of PRV information should be performed applying FFT and MUSIC algorithms, each of them with specific parameters for the selection of frequency resolution and interpolation of data. Future studies should aim to validate these results using real data and to evaluate how other technical aspects, such as the length of the recording and the presence of noise may affect frequency-domain analysis from PRV.

## Data Availability

The raw data supporting the conclusion of this article will be made available by the authors, without undue reservation.
